# Supplementary material for: Antibacterial Activity of Pharmaceutical-Grade Rose Bengal: An Application of a Synthetic Dye in Antibacterial Therapies
Source: Molecules. 2022 Jan 5;27(1):322. doi: 10.3390/molecules27010322 (PMC8746496; doi:10.3390/molecules27010322)
Supplement: Supplementary file 1 [file molecules-27-00322-s001.zip › molecules-1515870-supplementary.pdf]

molecules

Supporting Information

Article

Antibacterial Activity of Pharmaceutical-Grade Rose Bengal: An Application of a Synthetic Dye in Antibacterial Therapies

Michio Kurosu [1,*], Katsuhiko Mitachi [1], Junshu Yang [2], Edward V. Pershing [3], Bruce D. Horowitz [3], Eric A. Wachter [3], John W. Lacey, III [3], Yinduo Ji [2] and Dominic J. Rodrigues [3]

[1] Department of Pharmaceutical Sciences, College of Pharmacy, University of Tennessee Health Science Center, 881 Madison Avenue, Memphis, TN 38163, USA; kmitachi@uthsc.edu
[2] Department of Veterinary and Biomedical Sciences, University of Minnesota, 205 VSB, 1971 Commonwealth Avenue, St. Paul, MN 55108, USA; yang1181@umn.edu (J.Y.); jixxx002@umn.edu (Y.J.)
[3] Provectus Biopharmaceuticals, Inc., 10025 Investment Drive, Suite 250, Knoxville, TN 37932, USA; epershing@pershingenterprises.com (E.V.P.); bhorowitz@pvct.com (B.D.H.); wachter@pvct.com (E.A.W.); jlacey@utmck.edu (J.W.L.III); rodrigues@pvct.com (D.J.R.)
* Correspondence: mkurosu@uthsc.edu; Tel.: +1-901-448-1045

Table of Contents

Antibiotics ---S2
Cytotoxicity assays ---S3
Date for whole-genome sequencing of a PV-10 resistant *M. smegmatis* (ATTCC607™)—S4
References ---S4

Antibiotics

All antibiotics were purchased from commercial sources (amikacin disulfate salt (Sigma Aldrich, A1774-1G), capreomycin sulfate (Sigma Aldrich, C4142-1G), ciprofloxacin hydrochloride monohydrate (TCI, C2227), ethionamide (TCI, E0695), isoniazid (Sigma Aldrich, I3377-5G), linezolid (Chem-Impex, 29723), meropenem trihydrate (Ark Pharm, AK161987), rifampicin (Sigma Aldrich, R3501-1G)) and used without further purification unless otherwise noted. APPB (aminouridyl phenoxypiperidinylbenzyl butanamide) was synthesized according to the reported procedure. [74,75]

Cytotoxicity of PV-10 under light

Cytotoxicity assays for PV-10 in Vero cells were performed in a 24-well plate. Into each well (1 mL medium/well), 1 µL of drug concentration was added. After 1, 2, 3 and 4 h of incubation with drugs under the fluorescent light at r.t., the medium was removed and the cell was washed with PBS (x3). Images were collected by AMG EVOS xl transmitted light imaging microscope. After adding the medium (1 mL/well), 10 µL of MTT solution (5 mg/mL in PBS) was added and incubated for another 3 h at 37 °C (5% CO_2). The medium was removed, and DMSO (1 mL/well) was added. Viability was assessed on the basis of cellular conversion of MTT into a purple formazan product. The absorbance of the colored formazan product was measured at 570 nm by a BioTek Synergy HT Spectrophotometer.

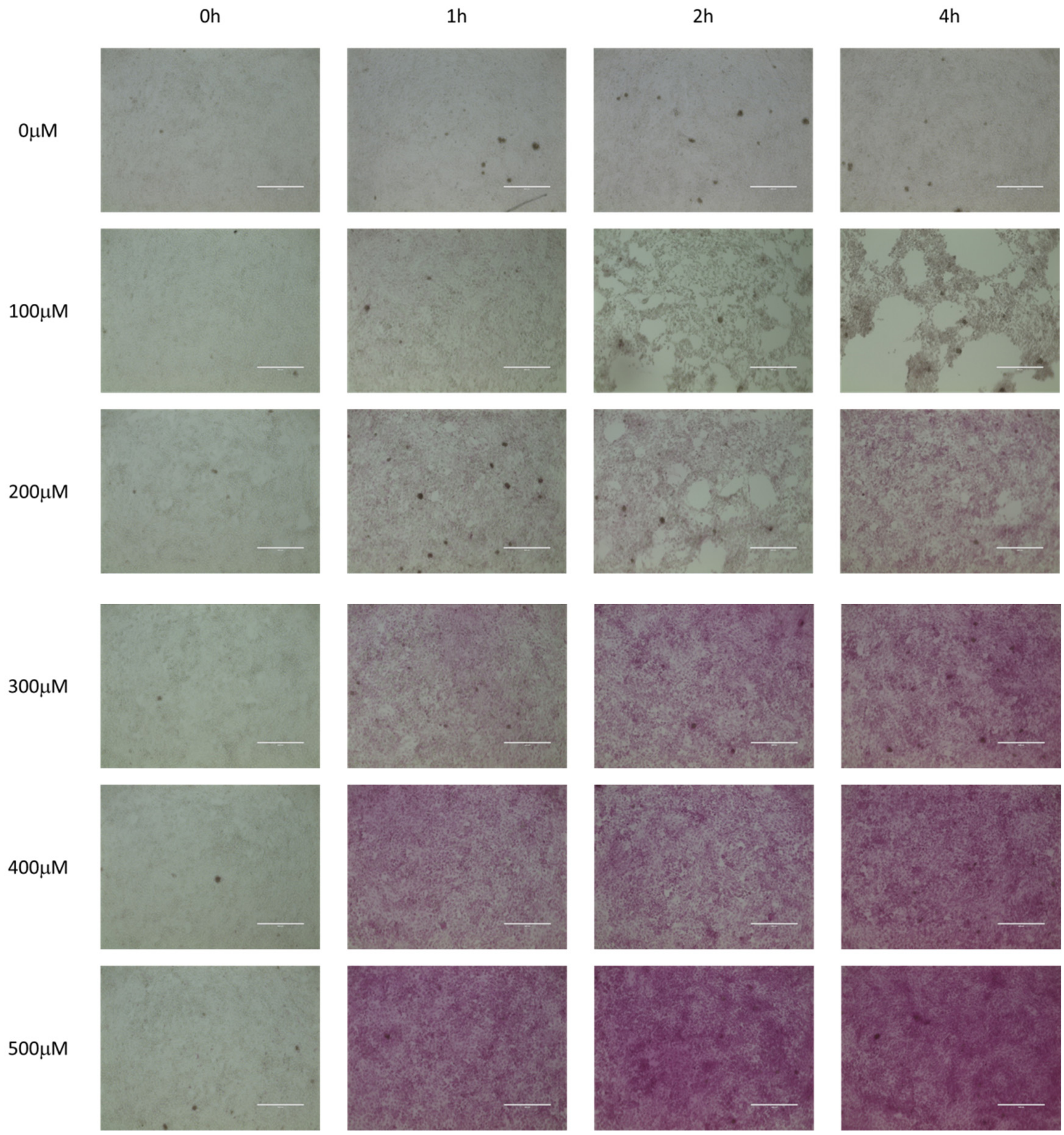

Figure S1. Top view images of Vero cells after PV-10 treatment. Scale bar = 400 µm.

Whole-genome sequencing of *M. smegmatis* strains

The procudeure was escribed in the tex (see section 3.11).

Table S1. The list of SNPs of M. smegmatis ATCC607 and its PV-10 resistamnt strain using *M. smegmatis* strain FDAARGOS_679 as a reference.

SNPs ATCC 607PvR and ATCC607 using Mycobacterium smegmatis strain FDAARGOS_679 as a reference

CHROM	POS	TYPE	REF	ALT	EVIDENCE	NT_POS	AA_POS	EFFECT	GENE	PRODUCT
NZ_CP054795.1	86860	snp	C	T	T:55 C:0			Nonsense		
NZ_CP054795.1	150471	snp	T	C	C:74 T:0			Nonsense		
NZ_CP054795.1	165620	del	GC	G	G:44 GC:0			Nonsense		
NZ_CP054795.1	179417	snp	C	G	G:94 C:0		430	GGG>GCG (Gly > Ala)	WP_011727516.1	UF2786 domain-containing protein
NZ_CP054795.1	209774	ins	C	CCTGGATGG	CCTGGATGG:29 C:0			Nonsense		
NZ_CP054795.1	252826	snp	T	A	A:100 T:0			Nonsense		
NZ_CP054795.1	278313	snp	A	G	G:79 A:0			GAT>GGT (Asp>Gly)	FOB87_RS01265	isopenicillin N synthase family oxygenase
NZ_CP054795.1	655132	snp	C	T	T:98 C:0			Nonsense	FOB87_RS03135	hypothetical protein
NZ_CP054795.1	697388	complex	TCGC	CCGT	CCGT:6 TCGC:0			Nonsense		
NZ_CP054795.1	697398	snp	A	T	T:5 A:0			Nonsense		
NZ_CP054795.1	750438	ins	C	CG	CG:69 C:1			CCG>CCGG)	FOB87_RS03560	hypothetical 750,139..750,539
NZ_CP054795.1	807713	del	TC	T	T:74 TC:0			Reading frame shift	FOB87_RS03885	molybdopterin-dependent oxidoreductase
NZ_CP054795.1	848667	snp	G	A	A:107 G:0			GGG>GAG (Gly>Glu)	FOB87_RS04055	hypothetical protein
NZ_CP054795.1	1278355	snp	T	C	C:102 T:0			TGG > CGG (Trp>Arg)	FOB87_RS06100	nuclear transport factor 2 family protein
NZ_CP054795.1	1314943	del	CG	C	C:92 CG:0			Reading frame shift	FOB87_RS06275	glycosyltransferase family 4 protein
NZ_CP054795.1	1322630	snp	T	C	C:87 T:1			GTC > GCC (Val > Ala)	FOB87_RS06315	1-acyl-sn-glycerol-3-phosphate acyltransferase
NZ_CP054795.1	2450878	snp	A	G	G:91 A:0			ATC > GTC (Ile > Val)	FOB87_RS11910	MMPL family transporter
NZ_CP054795.1	2463927	snp	A	G	G:100 A:0			Nonsense		
NZ_CP054795.1	2471229	del	GC	G	G:57 GC:0			Reading frame shift	FOB87_RS12000	diacylglycerol kinase
NZ_CP054795.1	2573215	snp	C	G	G:71 C:0			nonsense		
NZ_CP054795.1	2624938	snp	C	T	T:95 C:0			TGC > TAC (Cys > Tyr)	FOB87_RS12715	carboxylesterase/lipase family protein
NZ_CP054795.1	2636871	ins	A	AC	AC:81 A:0			Reading frame shift	FOB87_RS12770	hypothetical protein
NZ_CP054795.1	2646805	complex	GAT	CCGTC	CCGTC:62 GAT:0			Reading frame shift	FOB87_RS12830	DeoR/GlpR family DNA-binding transcription regulator
NZ_CP054795.1	3057956	snp	G	C	C:99 G:0			CGG > CCG (Arg > Pro)	FOB87_RS14690	IclR family transcriptional regulator
NZ_CP054795.1	3199404	snp	G	A	A:96 G:0			CGC > TGC (Arg > Cys)	murG	undecaprenyldiphospho-muramoylpentapeptide beta-N-acetylglucosaminyltransferase
NZ_CP054795.1	3231329	snp	A	G	G:98 A:0			CTG > CCG (leu > Pro)	ripC	peptidoglycan hydrolase RipC
NZ_CP054795.1	3324335	snp	A	G	G:72 A:0			ACG > GCG (Thr > Ala)	FOB87_RS15940	lipopolysaccharide assembly protein LapA domain-containing protein
NZ_CP054795.1	3886812	ins	T	TCGAGCGCGC	TCGAGCGCGC:78 T:4			(Leu Asp > Leu Gly Ala Leu Asp)	FOB87_RS18520	MBL fold metallo-hydrolase
**NZ_CP054795.1**	**4082258**	**ins**	**T**	**TG**	**TG:104 T:0**			**Reading frame shift at His71**	**rseA**	**anti-sigma E factor RseA**
NZ_CP054795.1	4087930	snp	G	C	C:95 G:0			AAG > AAC (Lys > Asn)	FOB87_RS19475	isoprenylcysteine carboxylmethyltransferase family protein
NZ_CP054795.1	4152291	snp	A	G	G:77 A:0			CTC > CCC (Leu > Pro)	FOB87_RS19770	nitrate reductase subunit alpha
NZ_CP054795.1	4307456	snp	A	G	G:86 A:0			CAC > CGC (His > Arg)	FOB87_RS20560	sensor histidine kinase
NZ_CP054795.1	4442430	snp	T	C	C:78 T:0			TAT > CAT (Tyr > His)	FOB87_RS21235	aminodeoxychorismate synthase component I
NZ_CP054795.1	4467112	snp	A	G	G:75 A:0			CGA > CGG (nonsense)	FOB87_RS21365	5-formyltetrahydrofolate cyclo-ligase
NZ_CP054795.1	4838982	snp	C	T	T:97 C:0			Nonsense		
NZ_CP054795.1	4853649	del	CG	C	C:100 CG:0			Reading frame shift	FOB87_RS23335	D-alanyl-D-alanine dipeptidase
NZ_CP054795.1	5060448	del	GC	G	G:74 GC:0			Reading frame shift	FOB87_RS24340	helix-turn-helix domain-containing protein
NZ_CP054795.1	5101901	snp	A	G	G:105 A:0			GTC > GCC (Val > Ala)	FOB87_RS24550	LysR family transcriptional regulator
NZ_CP054795.1	5205070	snp	T	C	C:97 T:0			Nonsense		
NZ_CP054795.1	5314448	snp	T	G	G:82 T:0			TGA > TGC (M >Cys)	FOB87_RS25575	ABC transporter substrate-binding protein
NZ_CP054795.1	5393330	del	GCCGAAAA	G	G:50 GCCGAAAAGC:0			Nonsense		
**NZ_CP054795.1**	**5717293**	**ins**	**G**	**GCACCCT**	**GCACCCT:71 G:0**			**Reading frame shift at Gly14**	**FOB87_RS27580**	**aquaporin family protein**
NZ_CP054795.1	5719323	del	ACGG	A	A:62 ACGG:0			GACGGC > GAC (AspGly>Asp)	glpK	glycerol kinase GlpK
NZ_CP054795.1	5719371	ins	A	ACATCCTCGGCG	ACATCCTCGGCGTCCCGGTGAGCAG:20 A			Iins	glpK	glycerol kinase GlpK
NZ_CP054795.1	5906171	snp	C	T	T:103 C:0			GCC > GTC (Ala > Asp)	recF	DNA replication/repair protein RecF
NZ_CP054795.1	5965160	snp	A	G	G:81 A:0			GAC>GGC (Asp>Gly)	FOB87_RS28670	hypothetical protein
NZ_CP054795.1	5990384	snp	G	C	C:78 G:1			CGG>CCG (Arg>Pro)	FOB87_RS28785	AAA family ATPase
NZ_CP054795.1	6193206	snp	A	G	G:70 A:0			SD region	fadD4	fatty-acid--CoA ligase FadD4
NZ_CP054795.1	6229399	snp	T	C	C:73 T:0			CGA>CGG(nonsense)	FOB87_RS29915	ferredoxin--NADP reductase
NZ_CP054795.1	6523244	snp	A	G	G:90 A:0			GTC>GCC (Val>Ala)	FOB87_RS31090	Crp/Fnr family transcriptional regulator
NZ_CP054795.1	6551108	snp	C	T	T:59 C:0			CCG>CCA(nonsense)	FOB87_RS31245	MSMEG_0570 family nitrogen starvation response protein
NZ_CP054795.1	6631566	snp	A	G	G:102 A:0			Nonsense		
NZ_CP054795.1	6673578	snp	C	A	A:98 C:0			CGG>CTG(Arg>Leu)	FOB87_RS31805	xanthine dehydrogenase family protein molybdopterin-binding subunit
NZ_CP054795.1	6735495	ins	G	GC	GC:92 G:0			Reading frame shift	FOB87_RS32090	XdhC family protein
NZ_CP054795.1	6836559	snp	A	T	T:82 A:0			TAC>AAC(Tyr>Asn)	FOB87_RS32600	Na+/H+ antiporter subunit A
NZ_CP054795.1	6844102	snp	G	A	A:56 G:0			Nonsense		
NZ_CP054795.1	6846422	snp	G	T	T:101 G:0			GCG>GAG (Ala>Glu)	FOB87_RS32655	AAA family ATPase
NZ_CP054795.1	6893010	snp	T	C	C:88 T:0			TGG>CGG(Trp>Arg)	FOB87_RS32850	DUF779 domain-containing protein
NZ_CP054795.1	6943702	snp	T	C	C:82 T:0			SD region	FOB87_RS33150	MspA family porin

References

74 Mitachi, K.; Yun, H-G.; Kurosu, S. M.; Eslamimehr, S.; Lemieux, M. R.; Klaić, L.; Clemons, W. M.; Kurosu, M. Novel FR-900493 Analogues That Inhibit the Outgrowth of *Clostridium difficile* Spores. *ACS Omega* **2018**, *3*, 1726-1739.

75 Mitachi, K.; Kurosu, S. M.; Gillman, C. D.; Yun, H. G.; Clemons, W. M.; Kurosu, M. A practical synthesis of a novel DPAGT1 inhibitor, aminouridyl phenoxypiperidinbenzyl butanamide (APPB) for in vivo studies. *MethodsX* **2019**, *6*, 2305-2321.
